# Active construction of southernmost Tibet revealed by deep seismic imaging

**DOI:** 10.1038/s41467-022-30887-3

**Published:** 2022-06-06

**Authors:** Zhanwu Lu, Xiaoyu Guo, Rui Gao, Michael Andrew Murphy, Xingfu Huang, Xiao Xu, Sanzhong Li, Wenhui Li, Junmeng Zhao, Chunsen Li, Bo Xiang

**Affiliations:** 1 Institute of Geology, Chinese Academy of Geological Sciences, Beijing, 100037 China; 2 School of Earth Sciences and Engineering, Sun Yat-sen University, Guangzhou, 510275 China; 3 Department of Earth and Atmospheric Sciences, University of Houston, Houston, TX 77204 USA; 4 College of Earth Sciences, Guilin University of Technology, Guilin, 541004 China; 5 Key Lab of Submarine Geosciences and Prospecting Techniques/Institute for Advanced Ocean Study, Ocean University of China, Qingdao, 266100 China; 6 Laboratory for Marine Mineral Resources, Qingdao National Laboratory for Marine Science and Technology, Qingdao, 266237 China; 7 Institute of Tibetan Plateau Research, Chinese Academy of Sciences, Beijing, 100029 China

**Keywords:** Tectonics, Geophysics

## Abstract

Southernmost Tibet exhibits an anomalously twice the normal thickness of average continental crust. There is no available theory to explain and the driving mechanism remains uncertain. Here, we interpret a north-striking, 180 km-long deep seismic reflection profile traversing the southern Lhasa terrane (SLT) to the central Lhasa terrane (CLT). In addition to reflections showing subducting Indian crust, our results reveal lateral heterogeneity between the SLT and CLT, where north-dipping reflections beneath the CLT outline a tilted crystalline basement, while the non-reflective domain beneath the SLT represents homogeneous juvenile crust. Our integrated analysis leads to models calling upon episodic magmatism onto the southern margin of the basement to result in progressive construction of the SLT. We hypothesize that this crustal thickening via crustal-scale magma accretion contributed to surface uplift of the southern margin of the Tibetan plateau and leading to the development of the vast internal drainage system of Tibet.

## Abstract (web summary)

Mechanism driving continental growth on modern Earth has long been debated. By interpreting the long deep seismic reflection data across the ongoing India-Eurasia collision zone, the authors report crustal-scale magma accretion onto the south of central Lhasa terrane produced net continental growth.

## Introduction

**Fig. 1:**
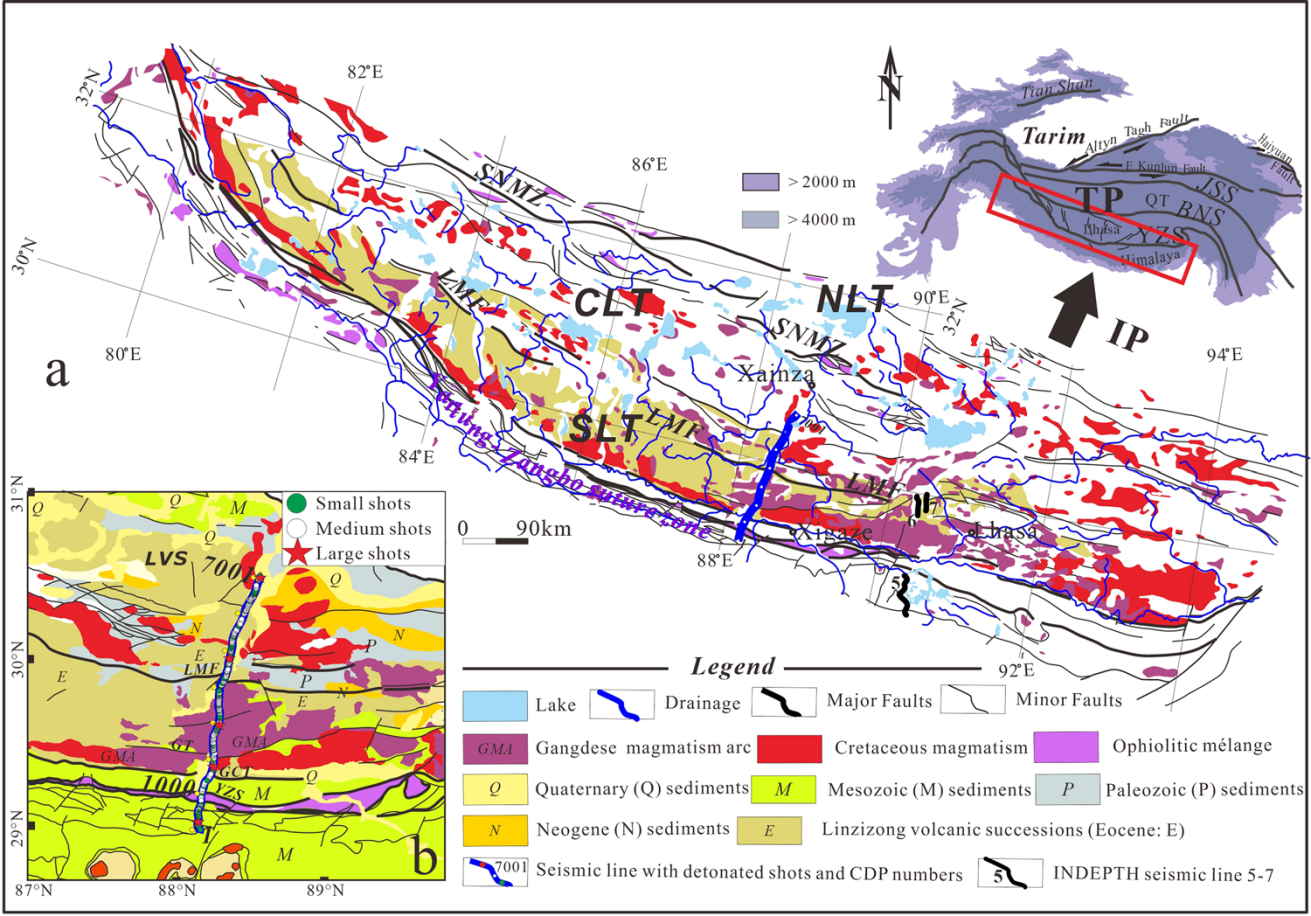
Geological map and research area. **a** Simplified geological map of the Lhasa terrane, southern Tibet (After China Geological Survey^48^). Numbered bold lines (5–7) indicate the INDEPTH seismic profiles that were deployed on both sides of the YZS; (**b**): zoomed-in map showing more details of surface geology of the research area. The inset shows the location of the research area (red box) in a larger context. Blue solid lines represent the location of seismic arrays with distribution of detonated shots and CDP numbers. TP the Tibetan Plateau, IP the Indian Plate, BNS Bangong-Nujiang suture zone, JSS Jinsha suture zone, NLT northern Lhasa terrane, CLT central Lhasa terrane, SLT southern Lhasa Terrane, SNMZ Shiquanhe-Namco mélange zone, LMF Luobadui-Milashan Fault, GCT Great Counter Thrust; GT Gangdese thrust, YZS Yarlung-Zangbo suture, LVS Linzizong volcanic succession, GMA Gangdese Magmatic Arc, See legend for more information of abbreviations (same abbreviations below).

The Lhasa terrane is located along the leading edge of the ongoing India-Eurasia collision (Fig. 1) and exhibits twice the normal thickness of average continental crust^1^. Its tectonic position is vital for not only being able to directly record the deep geodynamics of the collision but also being the chain connecting the Himalayan orogenic belt and the main body of the Tibetan Plateau to the north. The Lhasa terrane is therefore an ideal place for studying the mechanism driving crustal thickening of a converging domain.

Significant advances have been made on establishing the crustal thickening and uplift history of the southern Tibetan Plateau. The Gangdese Magmatic Arc (GMA) to the southern Lhasa terrane (SLT) (Fig. 1) is viewed as an Andean-type continental marginal arc that possesses normal continental crust prior to India-Eurasia collision^2–5^. The GMA has experienced active uplift to its present elevation since the onset of the India-Eurasia collision^2,6,7^. Meanwhile, geochemical analysis indicates the central Lhasa terrane (CLT) is lithologically composed of Precambrian microcontinent crystalline basement and the SLT consists of Phanerozoic juvenile magmatic materials^3,5^. Both sub-terranes have reached a double normal crustal thickness of ~78 km^1^. However, exact thickening processes remain disputed. Models in play can be categorized into three groups: (1) thickening by homogeneous pure-shear crustal shortening without crust-mantle material exchange^8^, (2) underplating of mantle-derived magma to an already thickened arc^2,9^, and (3) thickening by magmatic addition to an originally thin GMA crust prior to 45 Ma^10^. Groups 2 and 3 imply that the present-day thickness of the GMA crust, while located at the leading edge of the collision is largely the product of crust-mantle interactions. Although these groups of models are consistent with a variety of data, direct evidence of thickened crust by magmatic addition is lacking. Moreover, if valid it remains unclear how much syn-tectonic mantle input has contributed to thickening the crust, as well as how emplacement took place and whether there was mixing with the crystalline basement during magmatic fractionation.

The construction and present nature of the GMA crust is thus at the center of the debate. Juvenile GMA crust implies highly positive εHf (t) and positive ε_Nd_ (t) values^5^, which requires a pre-condition of either originally little crust beneath the GMA, or a large input of new material^10^ or both. Geochemical data are not able to constrain the crustal-scale distribution of the juvenile rocks, and although previous studies, e.g. INDEPTH, show clear crustal-scale architecture of the Himalayas to the south of our research area^11,12^, they lack detection capabilities necessary for imaging fine-scale crustal structure of southern Tibet. Therefore, what the spatial relationship between juvenile and ancient crust is and thus how the present crustal thickness was achieved remains uncertain. A better understanding of these issues is critical not only because the region defines the southern margin of the Earth’s largest continental plateau, but its development is hypothesized to be fundamentally responsible for creating the topographic barrier leading to the vast internal drainage system of Tibet and possibly reversed the course of the paleo Yarlung river^7^.

Present-day seismic reflection methods can differentiate large-scale, sub-surface architecture at high resolution^11^. This study reports results from a newly acquired 180 km-long, deep seismic reflection profile that crosses the southern and central Lhasa terranes. Structural interpretations help elucidate the fine crustal architecture and identify crustal anomalies. By integrating with previously established geophysical and geochemical constraints, we present a comprehensive model explaining the deformational history and processes leading to crustal thickening of southernmost Tibet.

## Results

### Geological background

The Lhasa terrane occupies southernmost Tibet (Fig. 1). It consists of three subterranes (Fig. 1), which include the southern, central, and northern Lhasa terranes. These are separated by the Luobadui-Milashan Fault (LMF) and the Shiquanhe-Nam Tso mélange zone (SNMZ), respectively^3^ (Fig. 1). The Lhasa terrane hosts extensive exposures of the Mid-Triassic to Miocene Gangdese Magmatic Arc (GMA) in the northern and southern Lhasa terranes and possesses ancient crystalline basement beneath the central Lhasa terrane^3,13^.

Genesis of the GMA complex is attributed to combined subduction of the Neo-Tethyan oceanic plate and subsequent collision of the Indian and Eurasian plates^9,13–15^. Lithologic trends in the GMA record late-stage subduction of the Neo-Tethyan oceanic slab^14,16^. Individual plutonic bodies document northward migration at 80–70 Ma followed by magmatic younging to the south at 70–43 Ma^14^. Meanwhile, the GMA is actively uplifting at rates similar to the Himalayas. GPS-derived vertical uplift rates show that the Gangdese batholith is uplifting at ~3 mm/yr^7^ and may be part of a zone of high topography propagating southward at an average rate of 5–8 km/Myr^10^. A shift in the magmatic trends at ~70 Ma^2,6,7^ indicates subduction roll-back of the Neo-Tethyan oceanic slab^14^. Breakoff of the oceanic slab is proposed to occur at *ca*. 52-51 Ma^14,16^, a process that is accommodated by another isolated intensive magmatic event in the GMA^14,16^. Additionally, an equal magmatic contribution has come from lower crustal partial melting after crustal thickening of southernmost Tibet during 26–12 Ma^17^.

Zircons extracted from the southern Gangdese igneous rocks in the southern Lhasa terrane exhibit relatively high εHf (t) and positive εNd (t) values, as well as low Hf crustal model ages (T_DM_^c^)^5,13^ (see Supplementary Information for explanations). Together with small mafic intrusions identified in granites of 65–41 Ma in age^9^, these isotopic characteristics indicate juvenile crust with mantle inputs during magma mixing and episodic emplacement^9,13–15,18,19^. The Gangdese batholith thus has experienced episodic magmatic underplating during partial melting of the mantle wedge and corresponding remelting of granitic rocks^5,9,13,20^.

### High-resolution crustal structure imagery

**Fig. 2:**
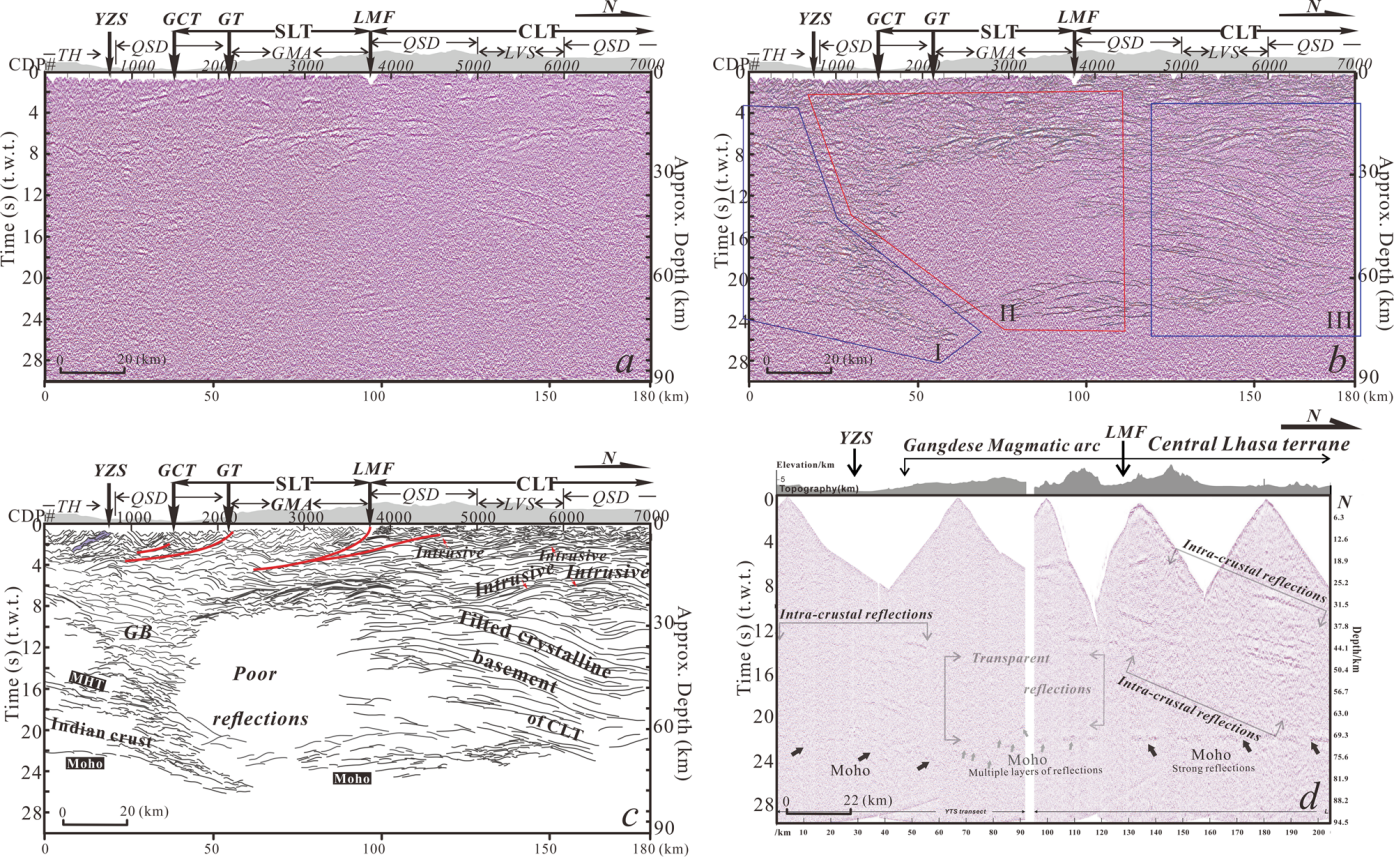
Deep seismic reflectivity data. **a** Uninterpreted 180-km-deep migrated seismic reflection profile (no vertical exaggeration) along 88.5˚ E. See Fig. 1 for location. **b** Superposition of composite line drawings of high-amplitude reflections. **c** Delineation of tectonic units in the fine-scale crustal transverse structure. **d** Single-shot section from the 2000 kg explosive source along the profile. Differentiation in intra-crustal reflections is observed from the south to the north, outlining three different domains within the tectonic converging zone. TH Tethyan Himalaya, QSD Quaternary sedimentary deposit, MHT Main Himalayan Thrust. See more abbreviations in Fig. 1.

Our seismic data (see locations in Fig. 1) was collected along a 100-km-long seismic reflection profile in 2015^21^ and a new 80-km-long profile in 2016. The profile transect runs perpendicular to the strike of major geological features in southern Tibet (Fig. 1). Figure 2a shows the high-quality migrated deep seismic reflection profile (see Supplementary Dataset S1 for higher resolution profile and Supplementary Information for acquisition parameters and processing steps).

The profile reveals highly reflective crustal structure down to depths of 26 s (t.w.t) (~78 km, assuming average crustal velocity of 6 km/s). Above 6 s (t.w.t) depth, reflectivity patterns correlate with surface geology (Figs. 1, 2). A sequence of south-dipping reflections coincides with the Yarlung-Zangbo suture zone, the Great counter thrust (GCT) and the Gangdese thrust (GT) (Fig. 2a). To the north, CDP 4000 coincides with the surface exposure of the Luobadui-Milasha Fault (LMF) (Figs. 1, 2a). Along with surface observations of E-striking thrust faults, seismic imagery connects the LMF with a sequence of reflection terminations that trace downward to merge with a band of high-amplitude reflection at ~4 s (t.w.t) (please see Fig. S2 in Supplementary Information for a zoomed in image). The seismic image and surface geology thus indicate that the LMF hosts the northernmost exposure of the GMA in the SLT (Fig. 2a, b). To the north of the LMF in the central Lhasa terrane (CLT), reflection terminations in shallow areas above ~4 s (t.w.t) of the seismic transect outline an anticlinorium (please see Fig. S3 in Supplementary Information for a zoomed in image), a structural feature that coincides with the Linzizong volcanic succession in the central Lhasa terrane. At further depths of 4–10 s (t.w.t), a sequence of convex-upward reflections is present (Fig. 2c). The surface geology along the seismic transect consists of extensive outcrops of GMA rocks. Since no large Cenozoic shortening structures exist along the transect we interpret these seismic reflections to represent regional igneous plutons^22^ at depths.

At intra-crustal scales, from south to north, the laterally distinctive reflection features and fabrics lead us to divide the entire seismic section into three parts (Fig. 2b, c). Zone I are from subducted Indian crust^21^. Zone II consists of the domain beneath the GCT and LMF of up to CDP 4000 (Fig. 2a, b). Surface geology between the GT and LMF shows exposures of the GMA (Fig. 1a). In the zone down to a depth of 10 s (t.w.t), reflections show a sequence of antiformal stacking geometries (Fig. 2c). Taking into consideration their location at the leading edge of the India-Eurasia collision, this feature may indicate a syn-collisional shortening and thickening of the GMA at a shallower depth via duplexing, a deformation style recognized from the northern Himalayas to southern Tibet^23,24^. At further depths below 8–10 s (t.w.t), the domain first shows a sequence of convex-upward reflections at ~8–10 s (t.w.t) and then is replaced with the appearance of a poorly defined to nearly non-reflective domain down to the base of the crust. The outline of this weakly reflecting feature indicates relatively homogeneous compositions that lack signal penetration beneath the southern Lhasa terrane^25^. Reflections within the southern segment of zone II in the paper by Guo et al. (2017) are non-reflective as well. It is difficult to find the impedance interfaces from them. This information supports a uniform crustal property of either crystalline basement or molten materials. The lower crust appears more seismically laminated nearby the CLT. Zone III occurs primarily beneath the CLT between CDP 4000 and 7000 (Fig. 2b, c). This zone exhibits relatively uniform deformation evident from a sequence of parallel, high amplitude reflections at depths of 8–22 s (t.w.t) (Fig. 2a, c). Together with previous geochemical studies^3^, these subparallel dipping reflections outline the crystalline basement beneath the central Lhasa terrane, which dip northward at depth (Fig. 2c).

The lower half of the seismic cross-section exhibits complex patterns with reflections of different lengths and dip angles (Fig. 2b, c). From south to the north, the base of the crust (Moho) is characterized by strong north-dipping reflections beneath the subducting Indian crust and becomes multiple layers of reflectivity beneath the SLT (Fig. 2b). A well-defined reflection appears at the base of the crust (at 22–26 s) between CDP 3000 and 5500 in the SLT indicating the position of the Moho as previous receiver function studies have shown^1^. The Moho geometry terminates nearby the domain beneath the CLT, where it is replaced in the vicinity of the Moho by a series of north-dipping reflections that root into the upper mantle (Fig. 2b).

In single shot, 2000 kg source datasets (Fig. 2d, 2000 kg shots shown as red stars in Fig. 1b), in addition to the three domains and their differentiation in crustal reflections, multiple layers of the Moho geometry in the migrated deep seismic section appear as well.

### Magmatic accretion

The entire deep seismic reflection image delineates the subducting Indian crust and overlying Lhasa terrane. The overlying crust displays lateral heterogeneity in crustal-scale seismic-reflectivity patterns, which are non-reflective beneath the southern Lhasa terrane (SLT) and north-dipping beneath the central Lhasa terrane (CLT). Non-reflectivity implies either no or slow impedance change of the medium^25^. The north-dipping structures record progressive deformation of the CLT, which is a dominant part of the original Lhasa terrane that possesses crystalline basement^3^.

**Fig. 3:**
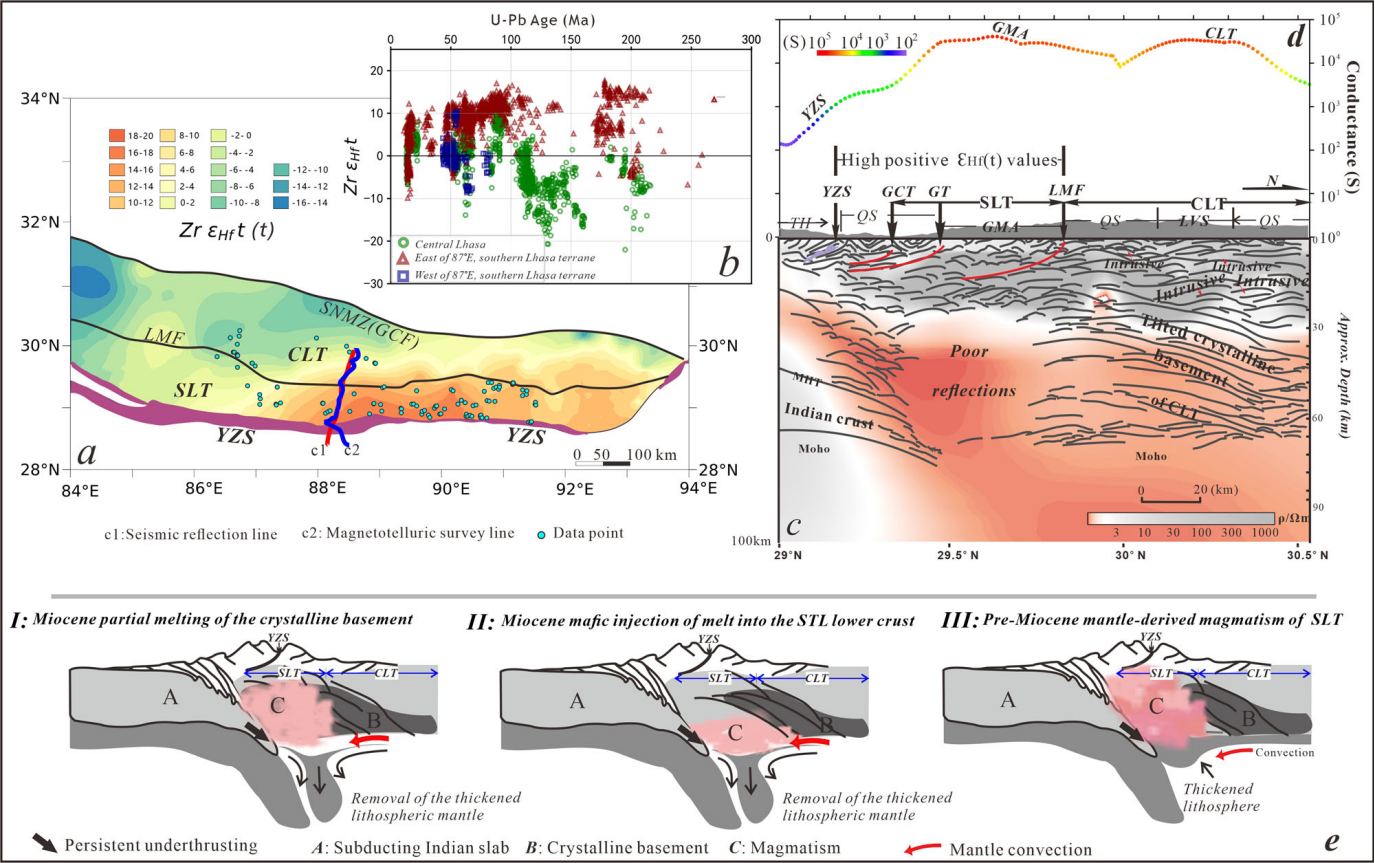
Integrated analysis. **a** Hf isotope contour map of the southern and central Lhasa terranes between of 84˚E and 94˚E showing the regional distribution of zircon ε_Hf_ (t) values as measured from Mesozoic-Cenozoic granitoids and felsic volcanic rocks. **b** Inset figure represents plots of zircon ε_Hf_ (t) values versus U-Pb age of zircons from felsic rocks. (Supplementary Dataset S2 lists literature sources for these values). **c** Comparison of the deep seismic reflection structures and the electrical structure by^27^ (see Fig. 3a for survey line location). **d** The electrical conductance values (in siemens (S)) calculated at depths ranging between 30–60 km by studies of Xue et al.^28^. **e** Three hypotheses proposed accounting for lateral heterogeneity in crustal-scale seismic-reflectivity patterns beneath the Lhasa terrane and the third style is the preferred model. See abbreviations in Fig. 1.

Zircon Hf-isotope data from Mesozoic-Cenozoic igneous rocks published over the last decade provide additional constraints on the juvenile nature beneath the SLT and crystalline basement beneath the CLT (Fig. 3a, b). A study by Li et al.^26^ from the Oligocene to Miocene rocks in the GMA indicate the εHf (t) isotopic values ranging between 16–20 generally imply a juvenile crust and few or no inputs of ancient crystalline basement. Meanwhile, available zircon U-Pb data and Hf-isotope data in Fig. 3b show the temporal-spatial variation in the southern and central Lhasa terranes. The results show the SLT to the domain east of 87° E have zircons ages of 15–120 Ma with predominantly positive εHf (t) values as high as 18–20. These scenarios, combined with the non-reflective crust, strongly suggests a different crustal property of the SLT from that of the CLT. Meanwhile, a nearly coincident electrical survey line was deployed along with the seismic reflection line^27^ (lines c1 and c2, respectively, in Fig. 3a). Integrating the results from these two studies show the non-reflective domain beneath the GMA has a high conductivity (Fig. 3c). The conductance that is calculated in association with the conductivity at depths between 30-60 km shows high values (≥10,000 S)^28^ (Fig. 3d). The melt fraction at the melt resistivity ranging between 0.1-0.3 Ωm beneath southern Tibet^29^ is constrained to values between 5% and 13% based on our calculation using the modified Archie’s law^30^. All the scenarios imply a partially molten, and thus relatively weaker crust of southernmost Tibet^29,31^, which may be driven by radiogenic heat generated from thickened crust or some mechanism else, such as thermal weakening from episodic magmatism.

The Moho defined along the crust-mantle boundary is generally viewed as a representative of the mafic residue left behind after partial melting of the lower crust and upper mantle^32^. Overprinting this “frozen” Moho requires significantly higher temperatures^33^. Thus, the multiple layers of Moho geometry beneath the STL strongly suggests that the Moho predates the ongoing partial melting, but probably postdates Miocene delamination of the lithospheric mantle throughout southern Tibet as the regionally identified peridotite xenoliths^34^ and adakites^17^ suggest contemporaneous intense crust-mantle interactions.

However, vertical interactions between crust and upper mantle will not result in the lateral differentiation in intra-crustal reflections between SLT and CLT. The north-dipping structures beneath the CLT (main part of the Lhasa terrane) are more likely associated with a combined shortening driven by both underthrusting of the Lhasa terrane beneath the Qiangtang terrane during Middle Jurassic^35^ and the northward subduction of the Neo-Tethyan oceanic slab during the Early Cretaceous^3^. A similar structural scenario is seen across the Trans-Hudson orogenic belt, where the lower crustal reflections appear parallel to the direction of Archean subduction^36^. Additionally, although previous geochemical/petrologic studies have greatly advanced our understanding of crustal features within the SLT, these methods are limited in delineating the crustal-scale structures. Moreover, existing tectono-magmatic models still view the SLT to be a part of the Lhasa terrane that possesses ancient basement^4,5^. Accordingly, we propose three explanations to account for the nature and deformation of southernmost Tibet (Fig. 3e): (1) the SLT and CLT share the same crystalline basement and Miocene partial melting “erased” the north-dipping structures beneath the SLT after removal of the thickened lithospheric mantle; (2) crystalline basement exists beneath the CLT and Miocene relamination^37^ of mafic igneous rocks took place into the lower crust beneath the SLT after lithospheric delamination; (3) crystalline basement exists beneath the CLT and, rather than Miocene magmatism, the SLT has experienced dominantly a pre-Miocene episodic magmatism. If the first hypothesis is valid, geochemical analysis should not reveal juvenile source magmas beneath the SLT, given the potential magmatic contribution from the ancient crystalline basement. For the second hypothesis, Miocene magmatism has problems explaining the origin of mixed magma between 50–70 Ma within the SLT. Meanwhile, the original nature of the crust beneath the SLT before Miocene partial melting is neglected.

Our synthesis leads us to the conclusion that the third hypothesis is the most viable explanation. Lateral heterogeneity in crustal reflections is due to differentiation in crustal properties between the SLT and CLT. The identified crustal architecture implies the SLT is originally thinner than normal continental crust or was part of the accretionary wedge that developed during oceanic subduction. The intense pre-Miocene episodic magmatism provided significant inputs leading to thickening the southern Lhasa terrane.

### Crustal thickening and surface uplift

**Fig. 4:**
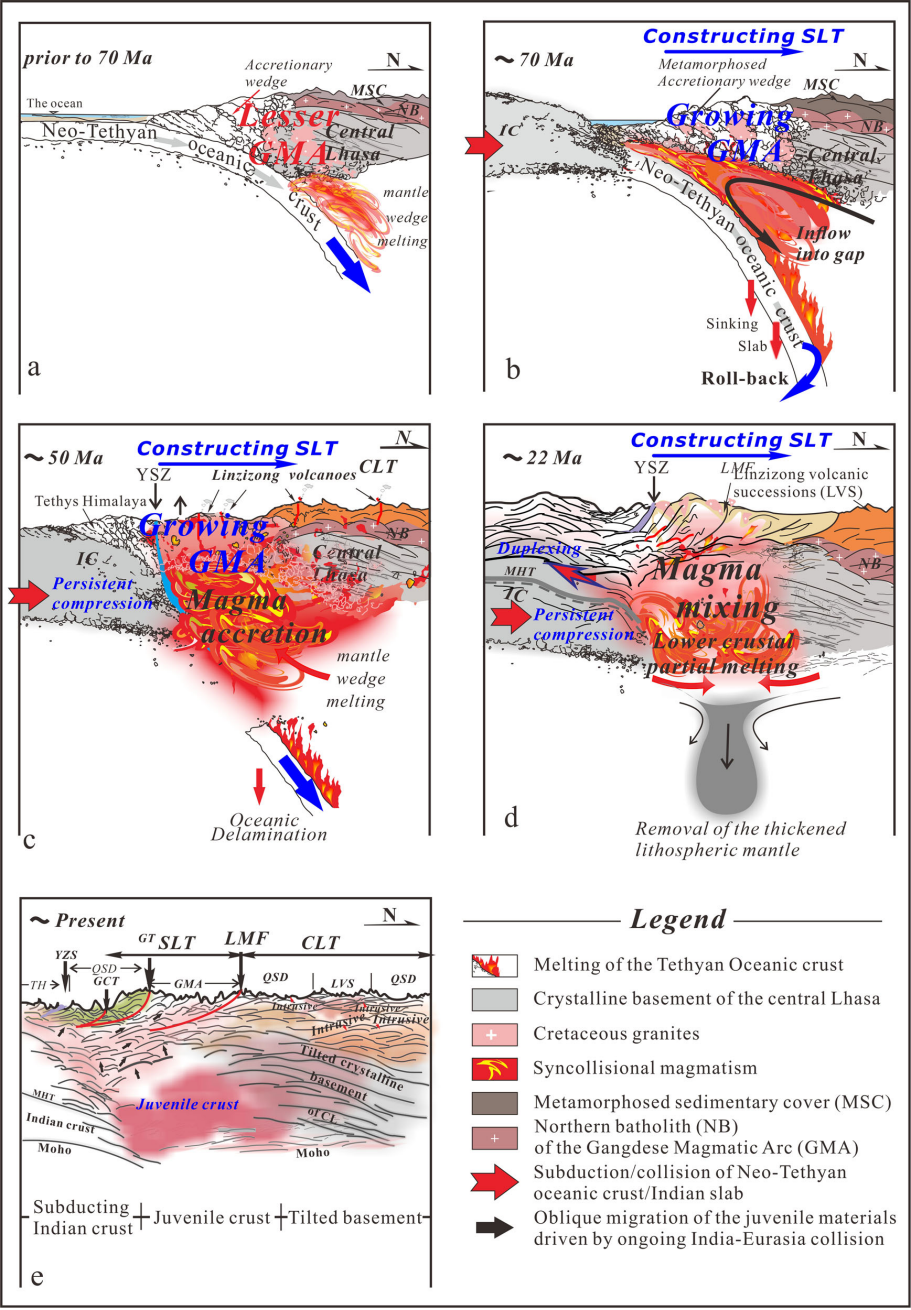
Continental Growth. Block diagrams (**a** to **e**) (not to scale) showing the episodic mantle input in generating juvenile crust. Each panel is based on ideas from previous studies. See text for details. Sketch of the accretionary wedge in the collision zone is inspired by^41,49^. See abbreviations in Fig. 1.

Our understanding of the origin of this correlation at depth is presented in evolutionary diagrams that build on previous studies^14,16,38^ and our interpretation of the crustal-scale architecture beneath the southernmost Tibetan Plateau (Fig. 4).

The evolution described begins prior to 70 Ma with northward migration of magmatism^14^ (Fig. 4a). Contemporaneously, combined with the amalgamation of the Lhasa and Qiangtang terranes during the Middle Jurassic^3^, the enduring northward subduction of the Neo-Tethyan oceanic slab beneath the entire Lhasa terrane^39^ led to the formation of the north-dipping structures in the central Lhasa crystalline basement that root into the Moho. Meanwhile, subduction delivered denser and metamorphosed portions of the oceanic slab to deeper depths, creating the potential for trench retreat^40^ (Fig. 4a). Subsequently, subduction roll-back at ~70 Ma defined by magmatic pulses, migrate to the south^14^ to occupy the gap that was created via trench retreat where they encountered the overlying, thickened accretionary wedge that is composed of a southward younging accretionary complex^41^ (Fig. 4b). In the SLT, the Dianzhong Formation of the LVS generated simultaneously to accommodate tectonothermal recycling with mélange-derived melt^42^ or partial melting of subduction-eroded crustal materials^4^. Slab break off of the Neo-Tethyan oceanic crust at ~52–51 Ma generated another major pulse of mantle-derived magmatic intrusion that took place via relamination in the form of diapiric ascent^37^ into the base of the lower crust of the SLT^14,16^ (Fig. 4c), which remelted the original juvenile crust to form granitic rocks. Figure 4d represents the latest post-collisional stage that features delamination of the thickened lithosphere and consequent relamination of mafic igneous rocks^37^ into the lower crust of the SLT^17,38^. Thereafter, multiple layers of the Moho geometry beneath the SLT were produced as a result of mafic sills from intensive mantle-derived melts, a scenario that is consistent with the double high-amplitude signals observed in the lower crust by analysis of broadband seismic data^1^. Additionally, the mantle-derived episodic magmatism within the juvenile SLT has probably experienced relamination to form the andesitic to dacitic GMA during crustal extraction^37^. Present-day (Fig. 4e), in addition to the pre-existing thermal weakness, the anomalous crustal thickness has probably generated more radiogenic heat resulting in high conductance values in the southern and central Lhasa terranes.

Therefore, in our model, heterogeneity in crustal reflections between SLT and CLT can be explained by episodic crustal-scale magma accretion onto the southern edge of the deformed crystalline basement. We envision this process resulted in lateral growth of southernmost Tibet and subsequent surface uplift that assisted in creating the vast internal drainage system of the Tibetan Plateau.

Isotope geochemistry and Trace/Rare Earth Element (T/REE) crustal thickness estimates paired with U-Pb zircon ages of GMA rocks provide additional constraints on the timing of arc magmatism and the paleodepth of the Moho from which these magmas were derived and had undergone fractionation. The last few years have seen notable attempts to quantify the paleo-crustal thickness of southern Tibet^2,10,43,44^. These studies exploit the observation that some pressure-dependent trace element ratios likely indicate the depth of melting in the mantle and therefore provide an estimate of crustal thickness^45^. Results from these studies in the SLT are consistent with an increase in crustal thickness of the GMA, possibly by as much as 40 km between ~60 Ma and 30 Ma. By 30 Ma the crustal thickness reached near present-day values as imaged in this study. Although crustal extension has been ongoing in the SLT since the Middle Miocene the observation that the crust has remained at the estimated thickness suggests that crustal thinning is balanced by crustal thickening probably by underthrusting of India as we advocate for in our model. Internal drainage of the Tibetan plateau is hypothesized to have developed by active uplift of the GMA where the modern drainage divide is located (Fig. 1a). Taylor, et al.^7^ further hypothesize that this uplift coincides with a reversal of the internal drainage flow from south-directed to north directed into the interior of the plateau. Although this uplift is interpreted to be driven by active duplexing as suggested by thermochronologic studies along the IYS^46^, we propose here that surface uplift during the Oligocene was driven in part by the active crustal construction of the SLT via magmatic addition/underplating.

The Zagros orogenic belt to the west of the Himalayan orogenic belt was produced during the ongoing collision between Eurasia and Arabia after closure of the Neo-Tethys Ocean, which possesses similar juvenile crust in the Urumieh-Dokhtar magmatic arc^47^. The tectono-magmatic evolution for creation of juvenile crust along the eastern segment of the Neo-Tethyan tectonic domain should therefore shed new light on understanding the net crustal growth along the western segment. Furthermore, the tectono-thermal evolution of southernmost Tibet provides a paradigm for understanding the crust-mantle interaction at convergent plate margins and consequent net crustal growth in collisional orogens of modern Earth.

## Supplementary information

Supplementary Information

Description of Additional Supplementary Files

Supplementary Dataset S1

Supplementary Dataset S2

## Data Availability

Uninterpreted deep seismic reflection profile used in this study is provided in Supplementary Dataset S1 and the compilation of published Hf-isotope data analyzed in Fig. 3b is provided in Supplementary Dataset S2.
