# Supplementary material for: Active construction of southernmost Tibet revealed by deep seismic imaging
Source: Nat Commun. 2022 Jun 6;13:3143. doi: 10.1038/s41467-022-30887-3 (PMC9170731; doi:10.1038/s41467-022-30887-3)
Supplement: Supplementary file 1 — Supplementary Information [file 41467_2022_30887_MOESM1_ESM.pdf]

SUPPLEMENTARY INFORMATION

Active construction of southernmost Tibet revealed by deep seismic imaging

Zhanwu Lu[1], Xiaoyu Guo [2*], Rui Gao[2], Michael Andrew Murphy[3*], Xingfu Huang[4*], Xiao Xu[2], Sanzhong Li[5,6], Wenhui Li[1], Junmeng Zhao[7], Chunsen Li[2], Bo Xiang[2]

1. Institute of Geology, Chinese Academy of Geological Sciences, Beijing, 100037, China
2. School of Earth Sciences and Engineering, Sun Yat-sen University, Guangzhou, 510275, China
3. Department of Earth and Atmospheric Sciences, University of Houston, Houston, Texas, 77204, USA
4. College of Earth Sciences, Guilin University of Technology, Guilin, 541004, China
5. Key Lab of Submarine Geosciences and Prospecting Techniques/Institute for Advanced Ocean Study, Ocean University of China, Qingdao 266100, China
6. Laboratory for Marine Mineral Resources, Qingdao National Laboratory for Marine Science andTechnology, Qingdao 266237, China
7. Institute of Tibetan Plateau Research, Chinese Academy of Sciences, Beijing, 100029, China

Corresponding author: Xiaoyu Guo guoxy37@mail.sysu.edu.cn

Mike Murphy mmurphy@central.uh.edu

Xingfu Huang huangxingfu@glut.edu.cn

List of contents:

1. Explanation of geochemical methods
2. Spreadsheet file is provided separately for a compilation of published Hf-isotope data for more accessible
3. Table S1: Acquisition parameters of the deep seismic reflection profile
4. Table S2: Basic steps for processing of the deep seismic reflection data
5. Figure S1: Uninterpreted deep seismic reflection image (provided separately due to its large size)
6. Figure S2: A zoomed-in image showing a close-up view of the crustal structures of the Luobadui-Milashan fault
7. Figure S 3: A zoomed-in image showing a close-up view of the crustal structures of anticlinorium above 4 s (t.w.t)

1. Explanation of geochemical methods

Analysis of Neodynium and Hf isotopes can be employed to identify the nature of the basements and ages of the continental crust (DePaolo and Wasserburg, 1976a, b; Dickin, 2018; Griffin et al., 2002)

(1) Hf isotope ratios

To trace evolution of crust and mantle, Hf isotope ratios are expressed as epsilon notation, which represents the measured $^{176}Hf/^{177}Hf$ ratio of the samples in terms of their deviation from thechondritic meteorites (CHUR) evolution line in parts per 10, 000 (Wu et al., 2007)

$\varepsilon Hf\ (t) = ((^{176}Hf/^{177}Hf)\ S-(^{176}Lu/^{177}Hf)_{S}\ x\ (e^{\lambda t}-1))/((^{176}Hf/^{177}Hf)_{CHUR,0}-\ (^{176}Lu/^{177}Hf)_{CHUR}\ x\ (e^{\lambda t}-1))-1)\ x\ 10000$ (1)

High positive εHf (t) values usually represent juvenile crust with mantle sources, while negative εHf (t) values indicate sources from ancient continental crust (Kemp et al., 2006).

(2) Nd isotope ratios

Nd isotope ratios (DePaolo and Wasserburg, 1976a, b) are expressed as epsilon notation that represents deviation of the measured $^{143}Nd/^{144}Nd$ ratio from that of chondritic meteorites (CHUR) in parts per 10, 000 (Dickin, 2018)

$\varepsilon Nd\ (t) = ((^{143}Nd/^{144}Nd)\ S-(^{143}Nd/^{144}Nd)\ CHUR))/(^{143}Nd/^{144}Nd)\ CHUR))-1)\ x\ 10000$ (2)

Positive εNd (t) values indicate a direct source from a depleted reservoir, while negative εNd (t) values imply the dated sample contain a large fraction of re-melted basement (Dickin, 2018).

(3) Sm-Nd model ages

T_{DM} represents the Sm-Nd model ages. It is defined as the time taken from the $^{147}Sm/^{144}Nd$ and $^{143}Nd/^{144}Nd$ ratios of the sample in present day to the time when the $^{143}Nd/^{144}Nd$ ratio is consistent with that of the depleted mantle source (DM). Under certain conditions, it can be interpreted as the age of the crustal rocks separated from the mantle reservoir, i.e. the age of "crustal formation" (Dickin, 2018)

References

1. DePaolo, D., and Wasserburg, G. J. G. R. L., 1976a, Inferences about magma sources and mantle structure from variations of 143Nd/144Nd: Geophysical Research Letters,

v. 3, no. 12, p. 743-746.

2. DePaolo, D., and Wasserburg, G.J., 1976b, Nd isotopic variations and petrogenetic models: Geophysical Research Letters, v. 3, no. 5, p. 249-252.
3. Dickin, A. P., 2018, Radiogenic isotope geology, Cambridge university press.
4. Griffin, W., Wang, X., Jackson, S., Pearson, N., O'Reilly, S. Y., Xu, X., and Zhou, X. J. L., 2002, Zircon chemistry and magma mixing, SE China: in-situ analysis of Hf isotopes, Tonglu and Pingtan igneous complexes, v. 61, no. 3-4, p. 237-269.
5. Kemp, A., Hawkesworth, C., Paterson, B., and Kinny, P. J. N., 2006, Episodic growth of the Gondwana supercontinent from hafnium and oxygen isotopes in zircon, v. 439, no. 7076,p. 580-583.
6. Wu, F., Li, X., Zheng, Y.-F., and Gao, S., 2007, Lu-Hf isotopic systematics and their applications in Petrology: Acta Petrologica Sinica, v. 23, no. 2, p. 185-220.

Table S1: Acquisition parameters of the deep seismic reflection profile

Recording system	428XL	Near offset(m)	25
Geophone(Hz)	10	Seismic source(kg)	50(small shot)<br>200(medium shot)<br>2000(large shot)
Receiver group spacing(m)	50	Shot spacing (m)	250(small shot)<br>1000(medium shot)<br>50000(large shot)
Numbers of one receiver group	24	Shot depth(m)	30*1(small shot)<br>50*2(medium shot)<br>50*15(large shot)
Number of channels	720(small shot)<br>900(medium shot)<br>1400(large shot)	Sample interval（ms）	2(medium/small shot)4(large shot）
Far offset（m）	17975 (medium/small shot)69975 (large shot)	Fold	72 (small shot)

Table S2: Basic steps for processing of the deep seismic reflection data

Read 30 s SEG-Y data
Apply geometry
Data splicing
Trace editing
Tomography static: datum level=5500m, replacement velocity=5000m/s Noise attenuation
Surface consistent deconvolution: prediction step=20ms; operator length=240ms; Velocity analysis: CDP increment=20-40
Residual static correction: iterations=6
PSTM
Data output

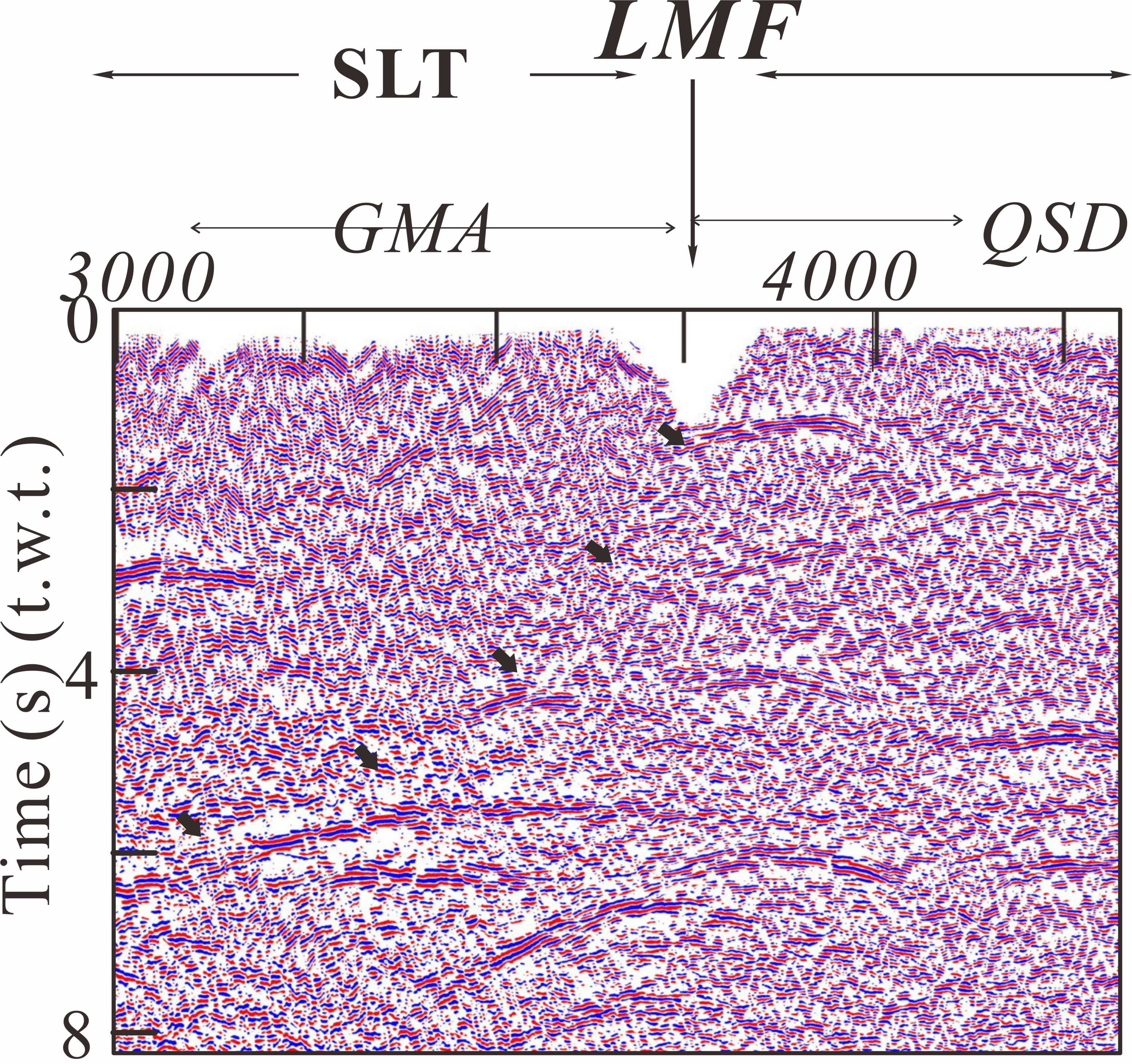

Figure S2

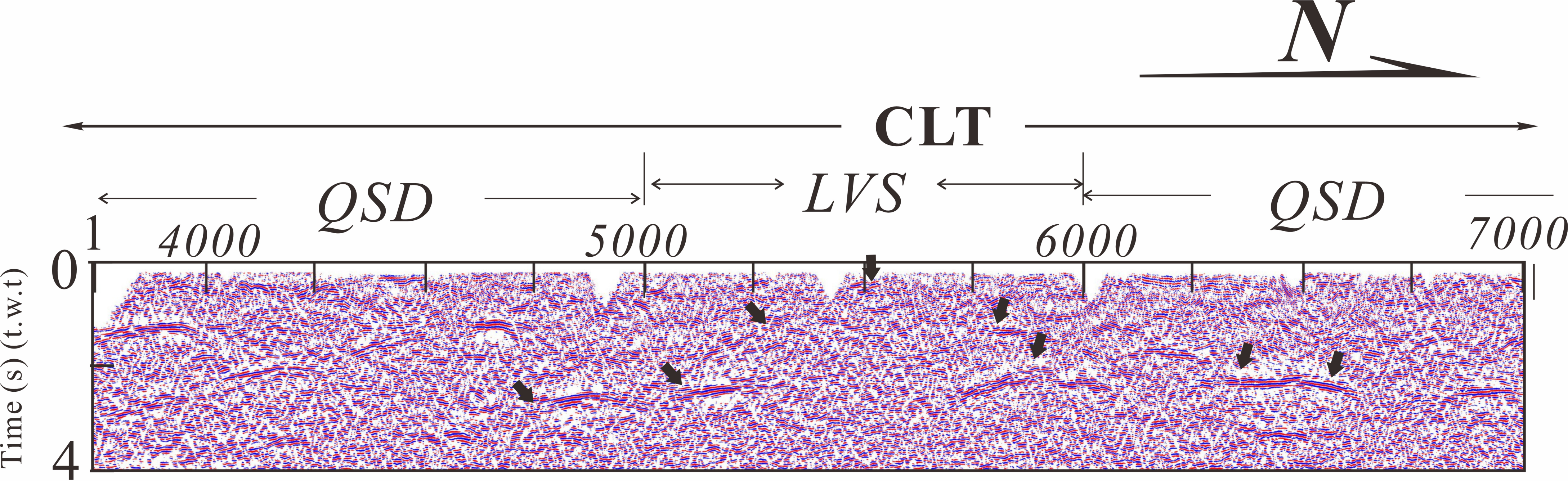

Figure S3
