# Supplementary material for: Active construction of southernmost Tibet revealed by deep seismic imaging
Source: Nat Commun. 2022 Jun 6;13:3143. doi: 10.1038/s41467-022-30887-3 (PMC9170731; doi:10.1038/s41467-022-30887-3)
Supplement: Supplementary file 2 — Description of Additional Supplementary Files [file 41467_2022_30887_MOESM2_ESM.pdf]

Description of Additional Suplementary files

File name: Supplementary Data 1
Description: Uninterpreted high-resolution deep seismic reflection image

File name: Supplementary Data 2
Description: Zircon Hf-isotope data from Mesozoic-Cenozoic igneous rocks published over the last decade
