# Supplementary figures and images for: Active construction of southernmost Tibet revealed by deep seismic imaging
Source: Nat Commun. 2022 Jun 6;13:3143. doi: 10.1038/s41467-022-30887-3 (PMC9170731; doi:10.1038/s41467-022-30887-3)

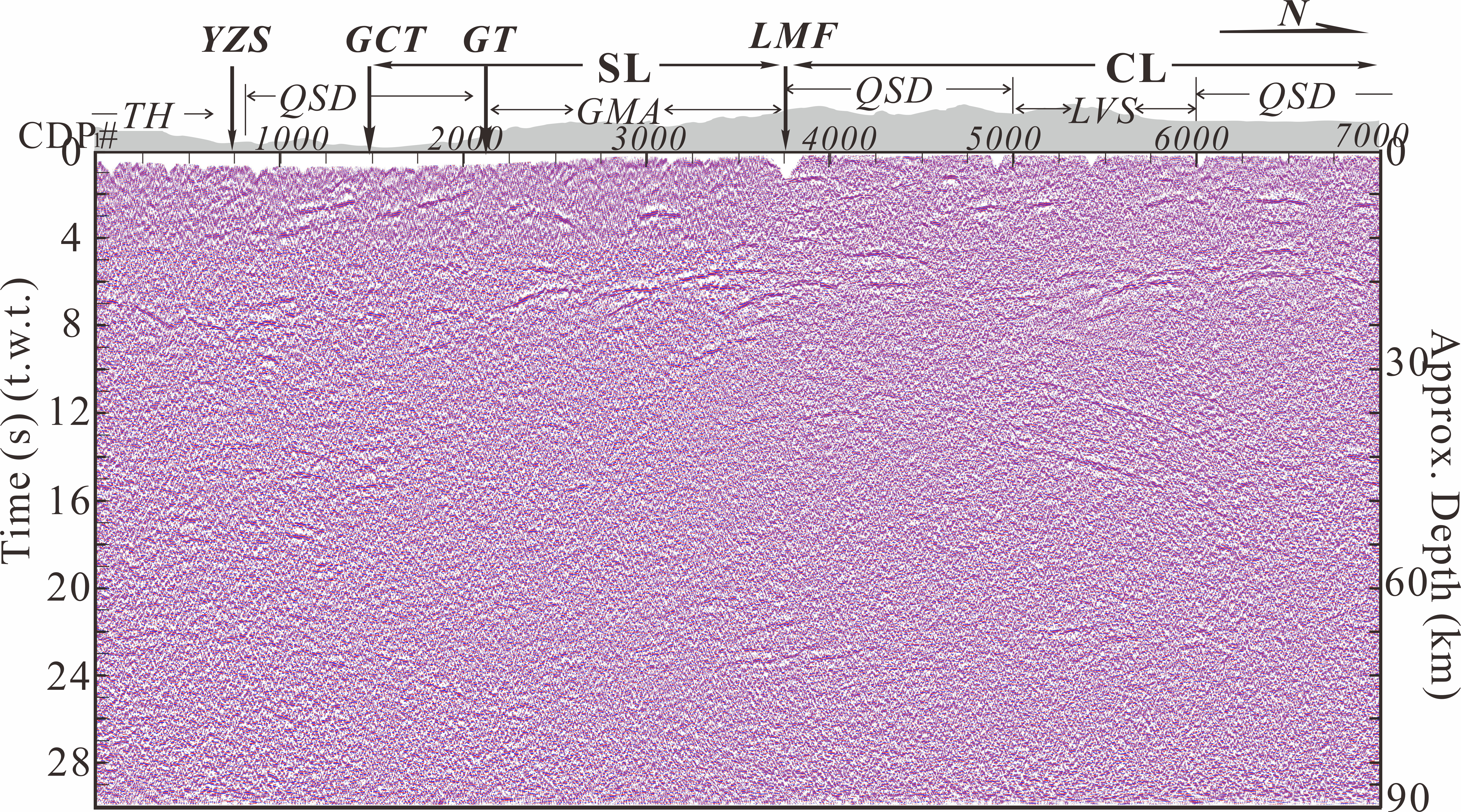

Supplement: Supplementary file 3 — Supplementary Dataset S1 [file 41467_2022_30887_MOESM3_ESM.jpg]
